# Bayesian Analysis of High-Throughput Quantitative Measurement of Protein-DNA Interactions

**DOI:** 10.1371/journal.pone.0026105

**Published:** 2011-11-01

**Authors:** David D. Pollock, A. P. Jason de Koning, Hyunmin Kim, Todd A. Castoe, Mair E. A. Churchill, Katerina J. Kechris

**Affiliations:** 1 Department of Biochemistry and Molecular Genetics, University of Colorado School of Medicine, Aurora, Colorado, United States of America; 2 Department of Pharmacology, University of Colorado School of Medicine, Aurora, Colorado, United States of America; 3 Department of Biostatistics and Informatics, Colorado School of Public Health, Aurora, Colorado, United States of America; University of Wyoming, United States of America

## Abstract

Transcriptional regulation depends upon the binding of transcription factor (TF) proteins to DNA in a sequence-dependent manner. Although many experimental methods address the interaction between DNA and proteins, they generally do not comprehensively and accurately assess the full binding repertoire (the complete set of sequences that might be bound with at least moderate strength). Here, we develop and evaluate through simulation an experimental approach that allows simultaneous high-throughput quantitative analysis of TF binding affinity to thousands of potential DNA ligands. Tens of thousands of putative binding targets can be mixed with a TF, and both the pre-bound and bound target pools sequenced. A hierarchical Bayesian Markov chain Monte Carlo approach determines posterior estimates for the dissociation constants, sequence-specific binding energies, and free TF concentrations. A unique feature of our approach is that dissociation constants are jointly estimated from their inferred degree of binding and from a model of binding energetics, depending on how many sequence reads are available and the explanatory power of the energy model. Careful experimental design is necessary to obtain accurate results over a wide range of dissociation constants. This approach, which we call Simultaneous Ultra high-throughput Ligand Dissociation EXperiment (SULDEX), is theoretically capable of rapid and accurate elucidation of an entire TF-binding repertoire.

## Introduction

Transcription is one of the most important control points for gene expression and is regulated in eukaryotes through multiple layers of control [1]. Sequence-specific DNA binding transcription factors (TFs) bind to specific genomic sites in promoters and enhancers, recruit additional proteins to achieve opening of chromatin, and ultimately assemble and activate RNA polymerase II pre-initiation complexes. Such transcription factor binding sites (TFBS) are essential to organismal function, yet there are substantial limitations in our current ability to predict the location and function of these elements [2]–[8]. In addition, mutations in regulatory sequences can easily alter transcription rates [9], [10], leading to the evolution of novel phenotypes, but the effects of TFBS mutations on transcription are generally unpredictable. This unpredictability exists partly because measuring TF-TFBS binding affinities in a high-throughput way remains problematic [11], and the number of large and accurate binding datasets remains few [12], [13]. To better understand the functional role of TF and TFBS interactions that contribute to altering gene regulation, methods capable of estimating such effects in a probabilistic and high-throughput manner are an important area of research. Here we focus on developing and testing a method that can be applied in a high-throughput fashion to estimate the binding affinities of TFs to their cognate TFBSs. Transcription is a process driven by the biophysics of these interactions, and thus they are often viewed as a necessary component to understanding transcription and biological networks [11], [14]–[24]. Our aim is that this Bayesian method should work for both strong and moderate binding relationships while taking into account the biophysical properties of these interactions.

A number of approaches have been developed to characterize details of how TFs bind to their cognate TFBSs. Established methods like SELEX and its high-throughput extensions [25] are, however, biased towards the highest affinity TFBSs. Other methods, relying on technologies such as electrophoretic mobility shift assays (EMSA), luciferase constructs, and proximity ligation analysis are labor-intensive or low throughput, and are typically used only to examine the affinity of variants of a known consensus binding site [26]–[29]. While protein-DNA binding microarrays (PBM) are a quite useful high-throughput technology that assesses binding of transcription factors to double-stranded DNA microarrays [22], [30]–[33] [31]–[33], PBMs make mostly qualitative measurements [18]–[24]. PBMs are also highly susceptible to inaccuracies due to loss of weakly bound material [11], while more accurate microfluidic devices [11] rely on specialized equipment. Other popular methods utilize chromatin immunoprecipitation (ChIP) followed by microarrays (ChIP-chip) to provide low resolution information on in vivo transcription factor occupancy, and this approach has been successfully employed for most of the known yeast TFs [34], [35] using microarrays of all intergenic regions. Higher resolution methods are now available based on ChIP followed by high density tiling arrays [36], by sequencing of DNA using paired-end diTAG, ChIP-PET, [37] or by direct next generation sequencing of ChIP products, [38], [39]. Limitations of ChIP-based methods are, however, that condition-specific protein binding can result in low enrichment and that it can be difficult to distinguish direct from indirect binding [22].

To generalize and extend characterization of measurement-based estimates of TF and TFBS affinities, position weight matrices (PWMs) have been used to summarize binding preferences so that new sequences can be scored for their potential to bind a specific TF. The PWM is based on nucleotide frequencies at each position, assuming each position contributes independently to the overall binding energy of the DNA-protein complex [40]. This approximation is correct when the TF is at extremely low concentration. The seminal work of Berg and von Hippel [41] related biophysical models of binding to the information content of the nucleotide frequencies used to construct the PWMs. Since then, biophysical models for DNA-protein interactions have been used to estimate binding affinities for a TF to genomic sequences given a PWM [42]–[46]. Several methods have been developed to estimate the energy terms in the biophysical model directly through high-throughput studies such as ChIP-chip and PBM [13], [47]–[50]. Many of the earlier methods assume low protein concentration [48], [50] for estimation or saturated occupancy [47]. More recently the BayesPI [49] and BEEML [13] methods have introduced models without these constraints.

In this paper, we introduce what we call a “Simultaneous Ultra-high throughput Ligand Dissociation Experiment”, or SULDEX. To evaluate dissociation constants, this approach utilizes high-throughput sequencing to count the relative numbers of short synthetic duplex DNA segments (ds-oligos) in solution before and after binding to a transcription factor. The goal of SULDEX is to: 1) simultaneously measure the relative binding abilities of large numbers of ds-oligos; 2) construct a biophysical binding model that can predict the energies of binding (and therefore the dissociation constants); and 3) integrate individual count-based binding estimates with model-based predictions informed by the entire repertoire; this allows better predictions of binding affinities when the frequencies of particular ds-oligos are low (either before or after binding). The SULDEX method is a Bayesian approach in which we apply Markov chain Monte Carlo (MCMC) methodology to obtain full posterior distributions of our unknown quantities.

SULDEX is comparable to two recently introduced techniques, Bind-n-Seq [12] and HT-SELEX [13], which utilize similar information. In contrast to SULDEX, the Bind-n-Seq approach [12] is designed to obtain approximate binding motifs that were previously unknown. As we demonstrate below, the Bind-n-Seq experimental design leads to data with undesirable qualities in terms of accurately measuring dissociation constants or binding energies. HT-SELEX [13] has more similar goals to ours, but is a maximum likelihood approach, and does not allow flexible assessment of binding model accuracy in the way that SULDEX does. Also, in contrast to these existing approaches, we incorporate into our method a means of predicting ds-oligo frequencies in the pre-bound solution. This is often critical for accurate results as these frequencies can easily vary by many orders of magnitude, even in cases where the nucleotide synthesis was designed to create equal ds-oligo frequencies. Furthermore, our method allows incorporation of multiple reference ds-oligos with known binding energies to obtain accurate dissociation constants in the absence of a good energy model. We evaluate the utility of our method using data simulated for ds-oligos with known dissociation constants for the Leu3 and ArcA transcription factors [26], [28], and Zif268 data from a Bind-n-Seq experiment [12]. Our results suggest that our Bayesian method can be used to accurately and precisely predict TF-TFBS binding affinities across a broad range of binding specificities. In addition to developing and testing our method, we provide extended focus on the interaction of experimental design with theoretical considerations.

## Methods

### Transcription factor binding

The binding interaction between a transcription factor, 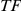, and a specific DNA sequence, 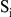, can be described by
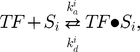
(1)where 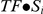 is the complex of 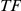 bound to 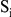, and 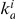 and 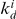 are sequence-dependent rate constants of association of the components and dissociation of the complex, respectively (also sometimes known respectively as 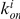 and 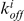). If 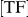, 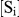, and 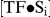 are the concentrations in solution of 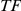, 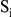, and 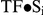, respectively, then at equilibrium, 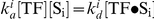 by definition. If we define the equilibrium constant 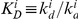, the proportion of the sequence that is bound at any point in time is given by
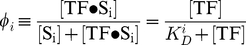
(2)This relationship is at the basis of most methods for determining 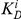. The 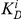 can be related to the standard free energies of binding, 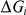, using the relationship 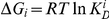, where 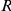 is the ideal gas constant and 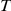 is the temperature (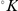). We note that this formula can be rearranged to derive a “chemical potential”, as in [13], [47], [51], but this is not necessary. Also, we do not assume that there is an unvarying non-specific binding component that is not specific to the sequence, as in [13].

In the SULDEX protocol, many sequences are mixed together in solution, and rather than measuring the proportion of a specific sequence bound, one measures the relative proportion of different sequences in both the pre-bound (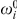) and the bound mixtures (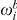). Thus, if 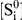 is the concentration of sequence 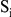 in the absence of transcription factors, and 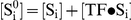 in the presence of a transcription factor, then we can estimate 
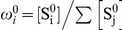
 and 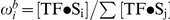, by sequencing and counting ds-oligos in the respective fractions. Note that we assume here (as is necessary in most experiments) that the frequencies in the bound fraction will not be affected by the purification process. This is probably a good assumption for strong binders, but will become problematic for weaker binders, for which 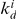 (known in this context as 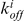) can be relatively large. We leave detailed consideration of this problem for future research.

We know that
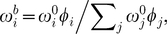
(3)so by substituting this into Eqn. 2 and re-arranging, we can see that if 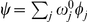 and 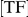 were known precisely, then the 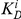 could be estimated as
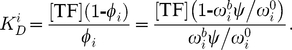
(4)It is worth pausing here to note that the probability of binding, 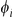, can in theory also be predicted using a partition function, as discussed by Stormo and colleagues [13]. In this context, Equation 3 has the same form as Equation 6 in that work, in which it is viewed as an application of Bayes' Theorem. We do not believe, however, that these two equations are exactly equivalent. One important difference is that here (as discussed in detail later) we model 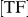 as a free (unknown) parameter. This allows us to avoid the issue of predicting 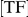 from first principles based on binding energies, conformations, and concentrations of the entire ensemble of sequences in the mix. This also leads to a second important difference, which is that given a known or inferred free 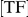, the probability that each sequence is bound does not depend on the other 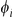. Another way of putting this is that the 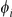 only affect each other through their effect on 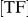. As a consequence, our Equation 3 should not be viewed as an application of Bayes' Theorem, but rather a statement of physical transformation such that the relative frequencies of molecules in the bound fraction will depend upon their relative frequencies in the pre-bound fraction multiplied by their probability of binding to the transcription factor, given the concentration of transcription factor available in solution at equilibrium. Furthermore, since this results in relative frequencies, the result of Equation 3 is a relative proportion among the sequences that have been chosen to be compared. In other words, 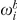 is not a posterior probability, but is instead formally a proportion among sequences considered, and the comparator sequences 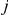 in Equation 3 and elsewhere may be summed over any desired subset of the sequences that were in the solutions.

In theory, 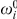 and 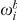 could be estimated as 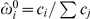 and 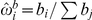, where the 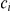 are the counts in the pre-bound fraction, and the 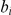 are the counts in the TF-bound fraction. Then, given two known reference 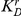, the two unknowns, 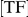 and 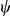, could be determined by solving the two ds-oligo-specific versions of Eqn. 3 (i.e., two equations with two unknowns). All of the unknown 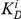 could subsequently be estimated using these values. In practice, however, it is preferable to take into account the uncertainty in 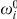 and 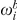. We chose to estimate all parameters using Markov chain Monte Carlo estimation under a Bayesian graphical model, described below, which can directly incorporate parameter uncertainty and allow that uncertainty to propagate properly through the conditional relationships. To reduce variance, it is also recommended that the reference sequences should be represented at a high frequency in the pre-bound fraction, and should be strong binders, so that they are highly represented in both the bound and pre-bound fractions. To produce exact reference dissociation constants in the basic binding model, at least two reference sequences are required. We note that with the incorporation of the energy model below, it is not strictly necessary to include reference sequences with known dissociation constants (as demonstrated by Stormo and colleagues[13]), although without reference dissociation constants only relative binding energies (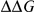
s) are produced. However, since our goal is to allow the results to depend more heavily on the count-based estimates of dissociation constants (or binding energies) than on the energy model (described below) in cases where the energy model appears to be unreliable, we have included reference sequences in all the analyses presented here, and highly recommend at least two whenever it is possible.

### A Bayesian graphical model for basic binding estimation

**Figure 1:**
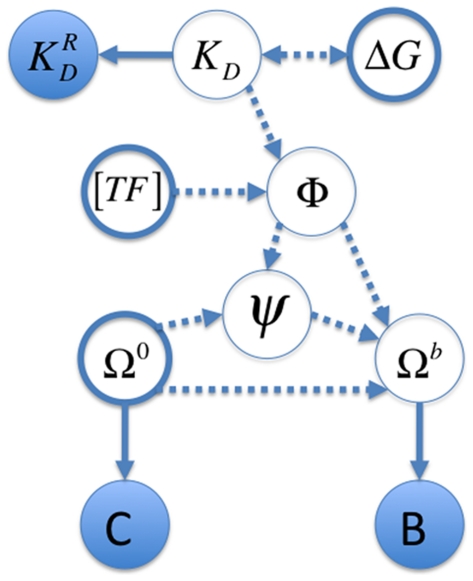
Graphical representation of the basic binding model (BBM). Observed variables (the pre-bound counts, 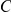, the bound counts, 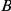, and the reference dissociation constants, 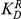) are in filled circles, and unobserved variables (i.e., parameters 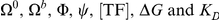) are in hollow circles. Probabilistic dependencies and their directions are shown with solid arrows, while deterministic dependencies are shown with dashed arrows. There is some flexibility in the graphical model as to which parameters are free and which are dependent on the others. In the implementations presented here, 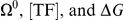 (thick bordered circles) were allowed to vary in the MCMC analyses, while 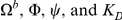 were calculated from the other parameters.

The goal of Markov chain Monte Carlo (MCMC) estimation is to estimate the posterior density 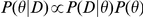, where 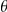 are the free parameters of a generative model and 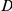 is the data. In the basic binding model (BBM), the data are the set of pre-bound counts 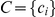 and bound counts 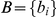, as well as a set of at least two (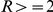) known reference dissociation constants 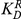 (Figure 1). The parameters are the transcription factor concentration in the bound solution 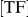, 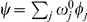, the set of pre-bound ds-oligo frequencies 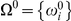, the set of bound ds-oligo frequencies 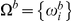, the set of binding probabilities 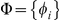, the set of dissociation constants 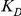 = {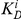} (not including the reference dissociation constants 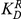), and the set of binding energies 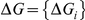. As frequency vectors, 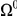 and 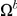 are constrained by 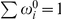, 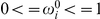, 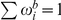, and 
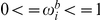
. Due to the dependencies described above and depicted in Figure 1, the sets 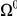, 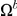, 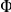, 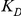 and 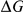, as well as the parameter 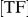, can all be calculated as functions of one another, and there is considerable potential leeway in choosing which parameters are free and which are not. In all implementations of the BBM presented here, we have chosen to model 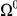, 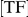, and 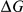 as free parameters, and calculated the remaining parameters as functions of these free parameters. We also note that it would not be difficult to include uncertainty about the reference constants (Figure 1), but we have not implemented this here.

The probabilities of the observed ds-oligo counts in the pre-bound and bound fractions are based on multinomials of the parametric frequencies of each ds-oligo in solution,
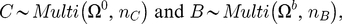
(5)where 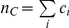 and 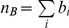. The log of the likelihood, 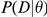, in the BBM is thus
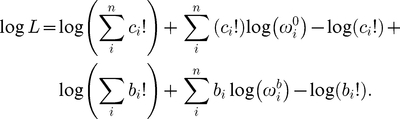
(6)As is clear from Figure 1, Equations 2 and 3, and our choice of free parameters, the 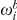 are calculated as functions of 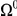, 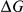, 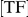, 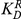, and 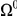.

We note that in this simple model, the main data-based confostraints on 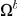 (other than the pre-bound and bound counts) arise from the set of known reference dissociation constants 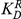 that directly affect only the two 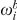 corresponding to the reference sequences. The only further constraint on the system is that the free transcription factor concentration in the bound fraction must be less than the total transcription factor concentration, if it is known from, for example, the amount of transcription factor added to the solution (i.e., 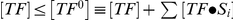). It would be straightforward, however, to add further boundary constraints or specify prior knowledge of any of the parameters (whether free or not). For example, the proportion of transcription factor in the unbound and bound fractions might be quantified, there might be error estimates on the reference dissociation constants, and some of the free energies of binding might have been previously predicted in some fashion (see below).

### A generative model of binding energy

It is reasonable to expect that in many cases the accuracy of estimating the frequency of the ds-oligos in the bound solution will be low (due to low counts). The accuracy of many of the calculated 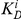 may also be low because they are much smaller than 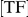, and therefore the probability of binding is close to 1.0 (see Equation 2). The accuracy of the 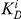 estimates will also be low if the 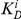 are much greater than 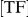, and therefore the probability of binding is close to zero. One potential way to improve the situation is to build a generative model of binding energies. It is also of general interest to better understand how binding energies are formed based on independent position effects and interactions among positions.

**Figure 2:**
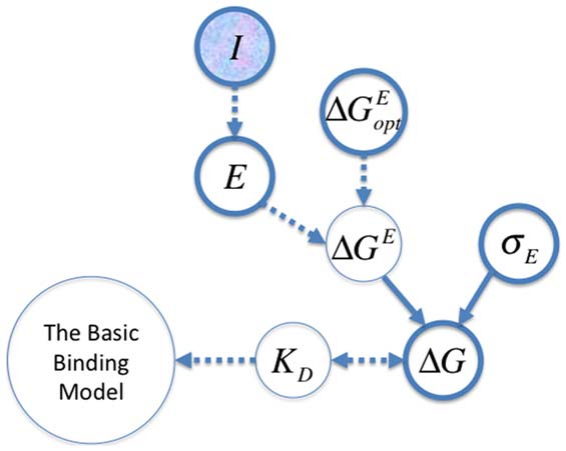
Graphical representation of the generative energy model (GEM). As in Figure 1, unobserved variables (i.e., parameters 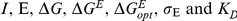) are in hollow circles, and probabilistic dependencies and their directions are shown with solid arrows, while deterministic dependencies are shown with dashed arrows. The Basic Binding Model (Figure 1) is linked as shown. As in Figure 1, the free parameters (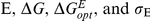) are shown with thick-bordered circles, as is the model indicator matrix, 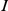. The dependent parameters calculated from the free parameters in the GEM are (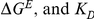), and the other parameters (not shown) are the same as described in Figure 1.

The general model we will consider to generate an energy-based 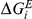 for a specific sequence 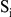 (Figure 2) includes positional energy terms relative to the optimal binding energy (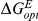); these positional terms are independent (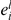) or interactive (e.g., 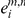), and the energy is given by:
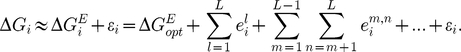
(7)Here, 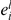 is an abbreviated way of indicating the position 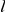-specific energy of the specific nucleotide (G, A, C, or T) that is found at position 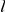 in sequence 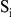. The independent position-specific energy terms are all zero (for the optimal sequence) or positive (since no sequence-specific binding energies can be lower than the optimum), and there are therefore up to 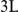 different independent energy term parameters. The abbreviation is similar for the pairwise terms, and the ellipses indicate the possibility of higher-order energy terms, although these were not implemented here. The interactive energy terms would be presumed to be zero unless otherwise justified, and constrained such that no sequence has a lower energy than 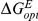. We use an indicator matrix, 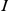, that controls the inclusion of individual energy terms in the model according to their posterior justification using a reversible jump Markov Chain (discussed in more detail below). We note that in the current implementation, the prior is uniform on each possible model. This may be more rigid than is desirable, and it may be a productive area for future research to consider other parameters, for example by including a hyperparameter on the probability that individual energy terms should be included.

In the examples here, we also include a random variable term, 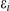, which allows for possible sequence-specific error in the generative energy model. Because the individual sequence-specific estimates are included as part of the GEM, this error term allows an automatic transition from the energy model predictions to the BBM predictions if the GEM is inaccurate or if the pre-bound and bound counts are especially accurate for a particular sequence. This variable is modeled as a normal distribution centered around zero and with variance 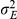. This is equivalent to stating that for the generative energy model (GEM),
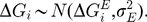
(8)Thus, 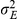 is a free parameter in the model that determines how much the GEM controls the range of credible sequence-specific energies (as opposed to control based on the observed sequence frequencies in the two solutions). We note that if only the additive portion of this model were used, the result would be similar to PWM scores only under the additional constraint of very low concentration of transcription factor [47], [52]. The log of the likelihood, 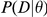, in the GEM is thus calculated as
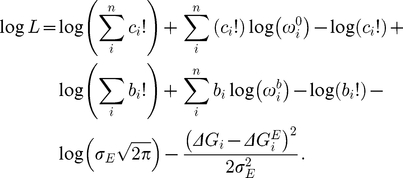
(10)As shown in Figure 1 and 2, 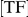, 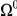 and 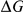 are still freely variable in the GEM. There are, however, two new free parameters in the GEM, 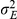 and 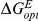, along with the new set of free energy parameters, 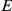, and the freely variable indicator matrix 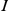 that controls inclusion of the various energy parameters in the family of GEM models. The dependent 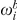 parameters in the likelihood calculations for the GEM model (Equation 10) are thus calculated using these new free parameters as well.

### A generative sequence model

**Figure 3:**
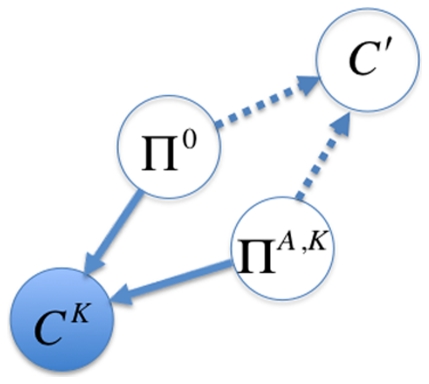
Graphical representation of the generative sequence model GSM. Symbols and arrows are as in Figure 1. The pre-bound counts, 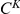, are given a superscript 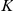 to indicate that they are counts for the shorter ds-oligos need for the GSM of size 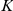. The parameter set includes the various polynucleotide probabilities used in the GSM (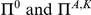) as well as the new predicted counts, 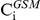. In the *SULDEX* program, the GSM can be linked to the BBM (Figure 1) using 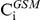 in place of C or 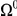, although that was not done in the analyses presented here due to problems with the available datasets.

In many instances, an experiment may be run in which there are too many ds-oligos synthesized relative to the amount of sequencing carried out, so that the accuracy of estimating the frequency of the ds-oligos in the pre-bound solution is low. In such instances, it may be advisable to build a Markov model of ds-oligo synthesis based on dinucleotide or higher polynucleotide observed frequencies in the pre-bound ds-oligo mix. We used a simple generative sequence model (GSM) whereby, if the ds-oligo was of length 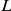 and the polynucleotide size was length 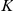, the GSM-predicted count, 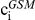, for a particular ds-oligo sequence 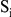 was
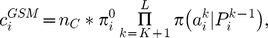
(11)where 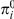 is the probability (in the entire data set) that a ds-oligo will start with the polynucleotide of length 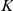 observed at the start of 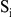, 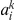 is the nucleotide observed at position 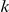 in 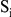, 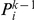 is the polynucleotide 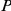 of length 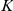 ending at position 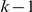 in 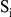, and 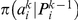 is the probability of 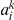 being observed in the data immefodiately following the observation of 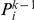. As before, 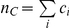 is the sum frequency of all observed full-length ds-oligo counts in the pre-bound fraction. The frequency 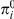 is included to take into account any beginning terminus bias in the sequence generation probabilities. The graphical representation of this model (Figure 3) therefore contains two input vectors that determine the sequence: the set of frequencies for all polynucleotides of length 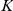 at the starting position 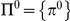; and 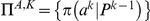, the set of probabilities for each nucleotide given that it is preceded by a particular polynucleotide of length 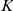. We note that we are treating these frequencies here as highly accurate estimates and not accounting for uncertainty in the estimates; the uncertainty should be negligible as long as 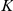 is small enough that the number of possible polynucleotides is much less than the total ds-oligo counts (i.e., 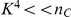). As will be clear in the results section, we did not utilize this model in any likelihood analyses in the current study based on our estimate of the lack of applicability of the model to current datasets. Instead, we evaluated the predictive utility of the model and used it to generate sequences for simulation of a full *SULDEX* study. However, the *SULDEX* program is designed to use 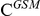 in place of 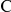 as needed, or in place of 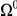 directly (foregoing the multinomial and not treating the 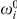 as free parameters) if 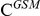 is believed to have negligible error.

### Markov chain Monte Carlo estimation

MCMC runs used the Metropolis-Hastings algorithm [53] acceptance ratio:
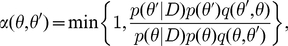
(13)where 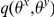 is the proposal density, or the probability of proposing a move to parameter set 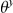 given a current state of 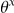. Symmetric (Gaussian) proposals were used throughout, so that the proposal ratios always cancelled out. Flat, non-informative priors were also used throughout, making the prior ratio also cancel. The acceptance ratio was therefore simply the ratio of likelihoods between the current and proposed parameter states, thus making the posterior distribution equivalent to the likelihood surface.

Proposal widths were tuned prior to each analysis, yielding an acceptance ratio close to the optimal value of 0.45 for unidimensional proposals [54] or around 0.234 for multidimensional proposals [55]. Analyses were run several times so that the ratio of within- to between-chain variances could be used to measure convergence (although in practice, running chains for 100,000 generations and excluding the first 10,000 as burn-in was generally adequate).

### Reversible jumps in model space

In GEMs, site-specific energy terms are set to zero (that is, they do not modify the optimum position-specific energy) unless there is strong evidence that a particular nucleotide at a position makes the binding worse. The vector of all energy terms is 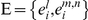. It is clear that only a limited number of interactive energy terms may be used in conjunction with the additive energy terms in order to avoid problems with non-identifiability of parameters. Here, we consider only the usage of the additive energy terms, and constrain all interactive energy terms to be zero. We also use an indicator matrix, 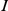, which tracks whether an element of 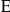 is constrained to equal zero in the current state of the model. When implementing the energy model, energy terms are added or removed (made equal to zero) using Green's reversible jump MCMC (RJMCMC) approach [56], which is generally used to switch between parameter spaces with different dimension sizes. All dimensional jumps increase or decrease the number of terms in E (and thus the dimension of the parameter space) by one, and these moves begin by either randomly sampling the value of a new energy term from a uniform distribution from 0 to width 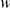, 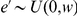, or collapsing an energy parameter term to zero. However, since these proposals may not be good samples near the optimum for the new dimension, we use a technique called “proposal reallocation” [57], in which we perform 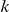 fixed-dimension MCMC steps through parameter space (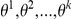) satisfying detailed balance with respect to 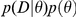. The final state 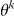 is then used for the decision to accept or reject the move to the new dimensional space, and we use a 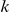 that is large enough (based on empirical tests) that 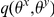 is assumed to be independent of 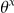, i.e., that 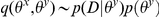. Note that we are thereby essentially using an arbitrary implicit prior ratio of 1∶1 for the two hypotheses, and that the ratio of time spent in any two adjacent dimensional spaces will be equal to the Bayes Factor between those spaces.

### Accuracy of the generative sequence model

For a given dataset, we evaluated the accuracy of the GSM for each polynucleotide of length 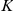 and ds-oligo length 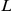 by comparing its predictions for frequencies of each ds-oligo 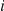 (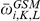) to their observed values. The observed values can be considered precise estimators for comparison (i.e., 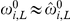) only in cases where the ds-oligos all have high counts, and in practice we could not go above ds-oligo length 7 for the GSM accuracy analysis. To evaluate estimator accuracy we used the root mean square error (RMSE), where 
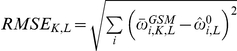
, which includes both bias and variance in the estimates as they deviate from the true value.

### SULDEX Simulations using existing control data sets

Two existing sets of data containing moderate numbers of 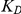
s were used for simulation analyses, 43 Leu3 [26], and 46 ArcA binding sites [28], with two reference sequences chosen for each data set. We also used the Zif268 Bind-n-Seq data (at 5 nM initial transcription factor concentration), including only the 436 ds-oligos that were within two nucleotide differences from the consensus binding site (GCGTGGGCGT). We used the top three best binders (GCGTGGGCGG and GCGTGGGAGG and the consensus) as reference 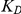
s (0.25728988, 0.579150579, and 0.15026296 nM, respectively). To determine the effectiveness of the SULDEX procedure in obtaining accurate dissociation constants and energy models, datasets were simulated based on known (or presumed) dissociation constants, mimicking the steps in a real SULDEX experiment. For simulations of the Leu3 and ArcA data, sequences were generated at equal frequencies. To avoid expected generation frequencies of zero for the Zif268 sequences, they were generated at frequencies equal to 0.8 times the observed frequency plus 0.2 times the frequencies obtained from a GSM with polynucleotide length 7. The accuracy of parameter estimates (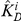) from the posterior means were then evaluated using the root mean square error (RMSE) of their logs, 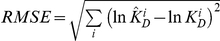. The protein concentration and the total copy number were varied in these simulations to determine what effect these experimental conditions have on the accuracy of the outcomes, and thus to guide experimental design. For the Leu3 and ArcA data, each 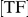 was categorized into low, medium, and high when its value was, respectively, lower than the lowest 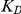, close to the lowest 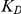, or in the middle of 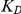 distributions of the target sequences.

## Results

In this study, we introduce multiple models for use in analyzing the results from SULDEX experiments. The first is the basic binding model (BBM), which can be used in situations where enough data has been collected such that most ds-oligo counts in the pre-bound and bound fractions are large enough to allow their frequencies to be estimated fairly accurately. We fit this model with the likelihood function given in Equation 6. The other two model classes are designed as extensions of the BBM. The generative sequence model (GSM) is designed to provide replacement estimates of counts in situations where many (or most) of the observed counts in the pre-bound fraction are zero or so small that they are estimated poorly. Such situations typically occur when there are exceptionally large numbers of sequences (e.g., 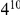 or 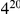 for length 10 or 20 random ds-oligos, respectively). As described below, however, we find that for available large-scale datasets neither the original counts nor this model are adequate, and so the GSM (Equation 11) was used only to generate synthetic datasets for testing the effectiveness of Bayesian analysis in determining parameters for the other models. Finally, we introduce the generative energy model (GEM) to provide better estimates in situations where some or many of the bound counts are not accurately determined. The GEM is actually not a single model, but a family of models in which various free energy parameter terms are turned on and off (included or removed from the model) during the course of the reversible jump Markov chain Monte Carlo analysis. We fit this model with the likelihood function given in Equation 10. The *SULDEX* program for implementing these analyses is freely available at the authors' web site, www.EvolutionaryGenomics.com. We begin the results with an analysis of experimental design considerations and the effectiveness of the GSM, followed by implementation and testing of the BBM and GEM on the small Leu3 and ArcA datasets, followed by testing of a full GEM-based SULDEX analysis of 10 mers simulated based on GSM count predictions from the Zif268 Bind-n-Seq 20 mer dataset.

### Experimental Design

An important consideration for experimental design is the synthesis of the ds-oligos that will be made into double-stranded targets and then bound to a transcription factor. This leads to several questions: What is the preferable target structure? What should the relative frequencies of different ds-oligos be? and what part of the binding site should be targeted with variation? SULDEX ds-oligos may contain primer regions for sequencing, variable sequence tags (or “barcodes”) to distinguish different experiments, and variable (experimental) and constant parts in the binding site and flanking region to prevent differential binding depending on which sequence tag is used. There is an inherent tradeoff between the goal of analyzing many sequences at once and the goal of obtaining accurate results. As accurate estimation of dissociation constants depends on accurate estimation of the pre-bound target frequencies, for a given number of sequence counts, including more ds-oligos in an experiment will lower the average number of sequence counts per ds-oligo. Furthermore, when many ds-oligos are included in the mixture, the variation in realized ds-oligo concentrations will mean that some ds-oligos are at much lower concentrations compared to others. For example, in the Zif268 data [12], hexamer (length 6) counts vary by 384 fold. In the HT-SELEX data [13], the expected ratio between the highest and lowest count 10 mer ds-oligo is over 1 billion.

This simple result has serious implications for designing experiments with satisfactory accuracy. One way to address this is to limit the number of ds-oligos per experiment. We can consider that if each ds-oligo we care about is sequenced 100 times in the pre-bound fraction, the standard deviation (std) of the count is 10% of its expectation (according to a Poisson assumption). Thus, we might want to sequence such ds-oligos 100–1000 times. It may be currently reasonable to expect 65 million reads in a single lane of Illumina HiSeq sequencing, meaning that one could reasonably target 65,000 different ds-oligos, hoping that with an average expectation of 1000 counts, a large fraction would be sequenced 100 times or more. For random ds-oligos, this means that only 8 sites should be varied (leading to 65,536 different ds-oligos). Although this may work for some studies, it may be unsatisfactory for others, and we suggest two alternatives to allow the entire binding site sequence repertoire to be evaluated at once. The first suggestion is to make smaller numbers of variants, and then mix them. For example, if five sites at a time were randomized in a 10 mer, and this was repeated 15 times over with a careful assortment of sites each time, then all pairwise deviations from the consensus sequence might be reasonably sampled in the mixture of these 15 syntheses containing 15,360 different ds-oligos. The second suggestion is to synthesize 50∶50 nucleotide mixtures at each site, focusing on those variants at each site that are thought to be relevant to binding the transcription factor in question. This is similar to a previously proposed approach for sampling ancestral sequences [58].

In cases where there are too many variants to get accurate relative frequencies in the pre-bound fraction, it is possible to use a predictive model. A simple model is to use the mononucleotide frequencies to predict ds-oligo frequencies, assuming that there is no higher-order interactive effect, as in [13]. A preliminary analysis of the Bind-n-Seq data [12] indicated that this simple model was not satisfactory, as demonstrated by the observation that the frequencies of many ds-oligos are substantially different depending on which direction they are considered. This is also true of the HT-SELEX data. We therefore developed an order and context-dependent generative sequence model (GSM) to include higher-order interactions using polynucleotides of varying length to predict subsequent sequences (see Methods).

It is straightforward to test the predictive accuracy of this GSM on shorter ds-oligos since the frequencies of short ds-oligos can be accurately measured by direct count. For the Bind-n-Seq data [12], the average predicted error for counts of 5 mer sequences is 1.96%, which is accurate enough to compare to GSM estimates. Using the observed values as a reference, the GSM with polynucleotide lengths of 1, 2, and 3 had RMSEs of 71.1%, 40.5%, and 22.1%, respectively. This indicates strongly that GSMs with longer polynucleotide dependencies are preferable, and thus that the longest polynucleotide that can be accurately measured (based on the predicted accuracy of the observed polynucleotide frequencies in a dataset) should be used. Although these predicted error rates are somewhat disappointing, the errors in predictions of 10 mer frequencies are likely to be considerably higher. Our sense is that there are other more complex trends in the data that may allow greater predictive accuracy, but that a greater number of independent synthesis datasets are required to determine if these trends are general and thus worth computationally pursuing.

The second step in SULDEX is to incubate the ds-oligo mixture with a transcription factor of interest. The concentration of free transcription factor in solution is the most important consideration in determining the outcome of the experiments (i.e., the accuracy of estimating binding energies or dissociation constants of interest). Unfortunately, this is difficult to predict ahead of time, as it depends on the concentrations and binding energies of all the sequences in solution, which are of course unknown at the outset. In cases where most of the sequences in the mixture are of interest (i.e., if variation in the mixture is targeted to be similar to the known binding motif), one can simply add different amounts of protein (holding the amount of sequence mixture constant), and from EMSA estimate the total ds-oligo content in the unbound and bound regions of tthe electrophoretic gel. An intermediate starting protein concentration can then be chosen in which approximately half of the DNA is in the bound fraction. Another possibility is to co-immunoprecipitate sequences bound to a transcription factor to measure the relative frequencies of the reference sequences in the pre-bound and bound solutions using quantitative PCR [59] or high-throughput sequencing.

### Analysis of the generative energy model with Leu3 and ArcA data

To demonstrate the utility of the GEM analyses, we simulated SULDEX sequencing experiments using pre-existing datasets of known dissociation constants for the transcription factors Leu3 and ArcA. Since the original data sets include dissociation constants for less than 50 distinct sequences, we could not generate enough information to evaluate interaction terms. Therefore, only independent position-specific (additive) energy components were included in analyzing these simulations. In general, it is unclear how accurately a simple additive GEM will estimate binding energies. As described below, additive GEMs estimated from the ArcA dataset predicted binding energies extremely well, although this was not true for the Leu3 dataset. The point of the GEM is to leverage the information about binding energies in the data set as a whole, to improve binding energy predictions for individual ds-oligos. If the GEM accurately represents the determinants of binding energy, then less sequencing may be required to achieve good results. An accurate GEM will therefore be particularly helpful for predicting binding energies for ds-oligos that are sequenced at low frequency, or that bind poorly, and are thus under-represented in the data. This insight motivated the inclusion of an error term (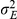) to account for inaccuracies in the energy model (see Methods). This error term is estimated along with the rest of the model, allowing an automatic transition from the energy model predictions to the basic binding model predictions when either the GEM is inaccurate or when large pre-bound and bound counts allow 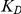
s to be accurately predicted by the BBM alone. When the counts get large, the model effectively factors apart into two subcomponents: a multinomial model in which all dissociation constants are entirely determined by the pre- and post-binding counts (and the reference dissociation constants), and an energy model that attempts to reproduce those nearly-certain dissociation constants in terms of a combination of energy parameters and error terms.

**Figure 4:**
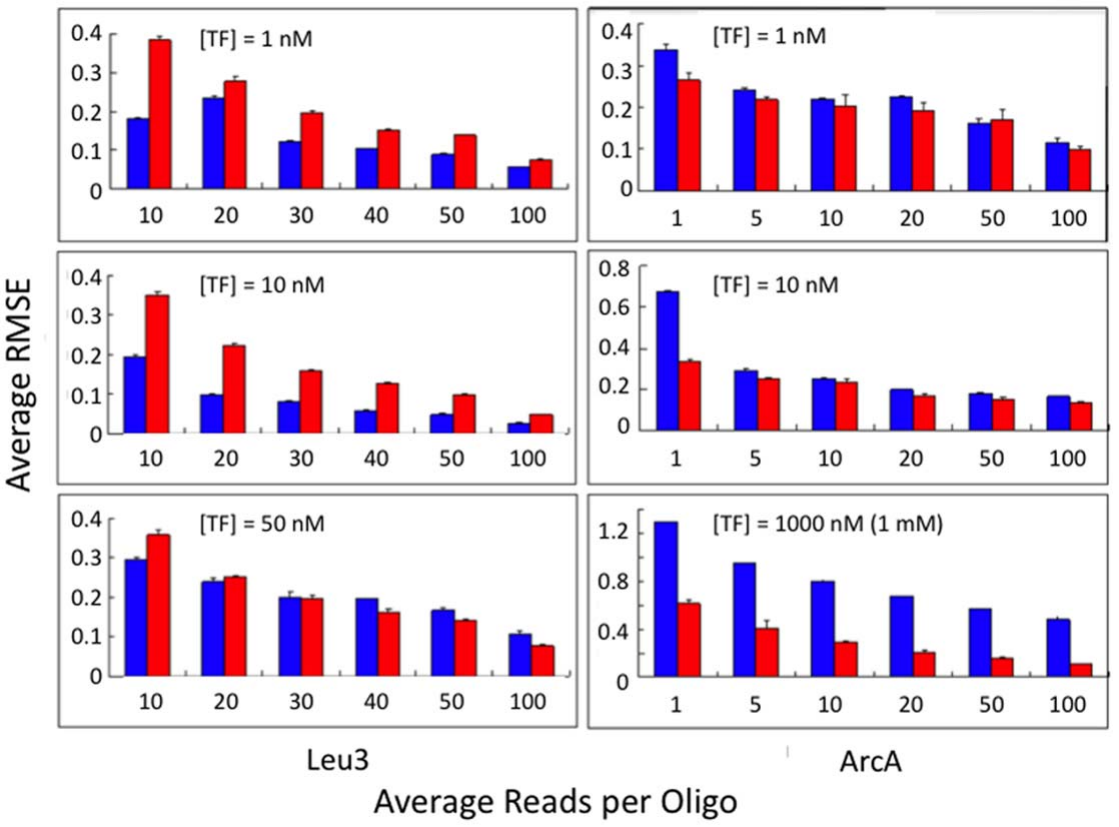
Comparison of the Basic Binding Model (BBM) and Generative Energy Model (GEM). The BBM results are shown in blue, and the GEM results in red. Data were synthesized based on the Leu3 (a) and ArcA (b) dissociation constant data sets. In silico sequences were equilibrated, resulting in 1 nM, 10 nM, or 50 nM free 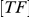 for Leu3, and 1 nM, 100 nM, or 1000 nM for ArcA. Values are the RMSE across all log 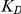 estimates, and error bars shown are for four replicates. Average reads (counts) per ds-oligo sequence ranged from 1 to 100.

As expected, binding predictions were more accurate (had lower log 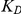 RMSEs) as the average number of sequence counts was increased (Figure 4). The results for Leu3 GEM analyses indicate that the error term improved predictions, since the RMSEs are mostly well below the value obtained when the error term was excluded (0.4). Although the GEM analytical predictions are not quite as accurate as the BBM predictions for Leu3, the error term allows the GEM to mostly reflect the accuracy inherent in the high counts and not the less accurate predictions of the energy model. For the experiments with Leu3 at a concentration of 50 nM, for which the BBM had the lowest accuracy among the Leu3 runs, there are some conditions for which the GEM analyses had slightly less error than the BBM analyses alone (Figure 4). Under these conditions (the highest transcription factor concentrations), many of the strong and moderate binders are almost completely bound (not shown), and it is therefore hard for the BBM to accurately distinguish the relative magnitude of their dissociation constants.

For the ArcA data, the GEM provides an improvement over the BBM in almost all cases. While the BBM RMSEs get substantially worse when the free transcription factor is high (Figure 4; note the differing scales on the graphs), the GEM RMSEs are similar for all but the lowest counts. The difference between the two methods is most notable in the results for the 1 µM (1000 nM) concentration of transcription factor. Evaluation of the errors as a function of 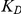 (Figures S1a and S2a) indicate that most of the error reductions come from the best or the worst binding ds-oligos under conditions where they are poorly estimated by the BBM. It is also worth noting that for ArcA, with its relatively accurate GEM, increases in the amount of sequencing only slightly alter the RMSEs under the GEM, and the variance of the error estimates are slightly narrower (Figure S2b). In contrast, the error terms for the Leu3 GEMs become considerably larger with increases in the amount of total sequence counts collected (Figure S1b). One way to view this is that as more counts accumulate, the BBM estimators become more accurate and thereby better expose the inaccuracy of the Leu3 GEM estimates. Thus, the change in the error estimate with increased sequencing seems to serve as an indicator of the reliability of the GEM.

### Relationship between posterior energy model terms and binding site consensus

Leu3 binds to a palindromic consensus site (CCGgtacCGG) as a homodimer to regulate genes involved in branched-chain amino acid metabolism [60]. There are 10 positions in the palindromic binding site, but since only 43 variants with known dissociation constants were available for the simulation analysis, there is a great deal of missing data. Positions 2 and 9, for example, do not vary, and there are only two variants each at positions 3, 8 and 10. In the original experiment [26], variants with mutations at positions 2 and 9 were too deleterious to Leu3 binding to be observed. An examination of the posterior mean binding energy estimates for the intermediate binding conditions (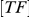 = 10 nM) and moderate counts (average 10 per ds-oligo) indicates that a number of positions have only marginal differences between the optimal variant and the next-best variant (Table 1). At position 3, for example, the mean posterior position-specific energy difference between G and T is only 0.03 energy units, and at position 7 the difference between C and T is only 0.04. In contrast, the nucleotide T at position 1 is the most deleterious variant, with 1.9 additional energy units difference from C, which is the optimal nucleotide at this position.

**Table 1:** Posterior distribution of position-specific energy terms for Leu3, 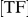 = 10 nM, average counts = 10).

	A		C		G		T	
Position	mean	std	mean	std	mean	std	mean	std
1	-	-	0.00*	**0.01**	0.32	0.21	1.88	0.46
2	-	-	0.00*	**0.00**	-	-	-	-
3	-	-	-	-	0.02*	**0.05**	0.05	0.10
4	0.91	0.45	0.15	0.19	0.04*	**0.10**	0.19	0.22
5	0.42	0.15	0.58	0.20	0.80	0.26	0.00*	**0.01**
6	0.01*	**0.03**	1.22	0.30	0.33	0.36	0.61	0.18
7	0.57	0.25	0.25*	**0.16**	0.71	0.51	0.29	0.47
8	0.28	0.27	0.02*	**0.07**	-	-	-	-
9	-	-	-	-	0.00*	**0.00**	-	-
10	-	-	0.92	0.35	0.00*	**0.01**	-	-

The lowest energy term at each position is in boldface, and the mean for the consensus sequence is indicated with a *. Variants that were not tested in the analysis have a dash.

**Figure 5:**
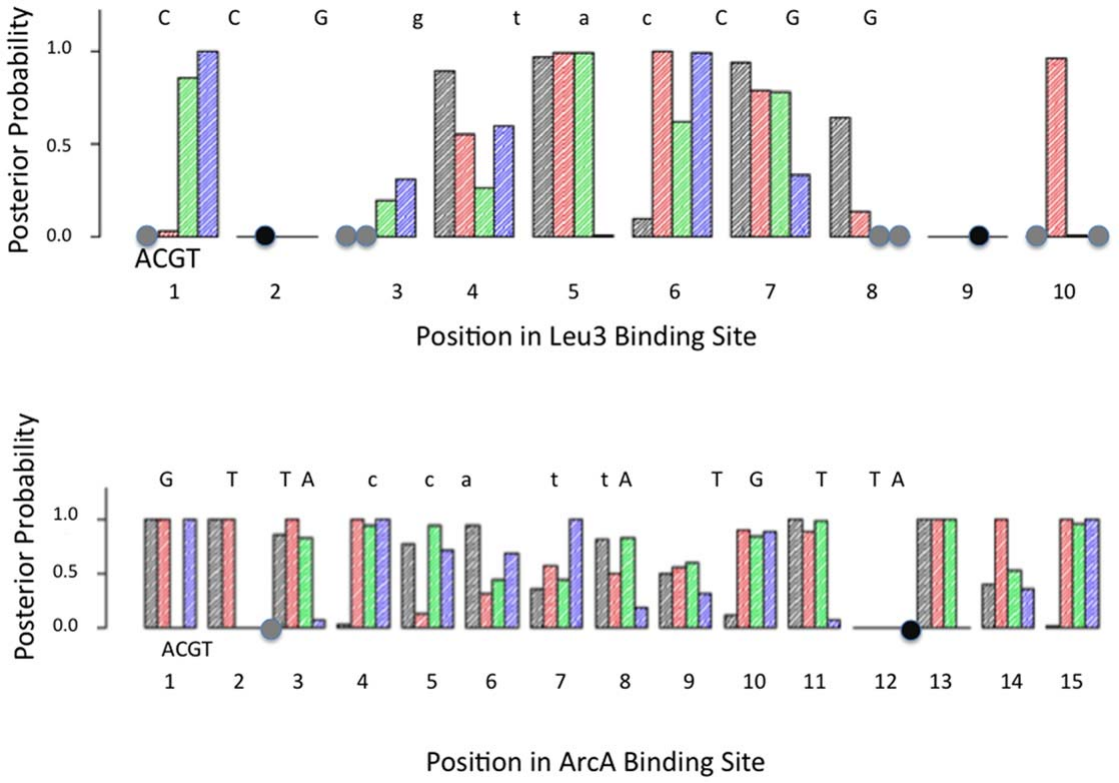
Posterior probability of position-specific energy terms in Leu3 and ArcA. The posterior probability for each nucleotide at each position is shown in the order A (black), C (red), G (green), and T (blue). Gray dots are shown for nucleotides for which no information was available in the data, and black dots are shown over the nucleotide used at a site where no variation was available at that site. The consensus sequence is shown above the probability bar corresponding to the consensus nucleotide. Capital letters indicate a strongly preferred consensus nucleotide at a position, while lower case letters indicate a more indeterminate consensus nucleotide. Conditions are specified in Tables 1 and 2.

One of the features of our modeling approach is that Markov chains are run over different models that include varying numbers of parameters. For example, an energy term might not be required if the corresponding variant is energetically indistinguishable from the optimal binding variant. In general for Leu3, a mean posterior energy difference of about 0.6 or greater is associated with a posterior probability of >0.95 that an energy term is required (i.e., that having that nucleotide at that position worsens the energy of Leu3 binding enough that an energy term is required to distinguish it from the optimal variant; Figure 5). As expected, the lowest energy terms at each position generally match the consensus (Table 1).

ArcA binds as a symmetric dimer to a tandem-repeated 15-position consensus site (GTTAccattATGTTA) to regulate genes involved in oxygen response [28]. There were 46 sequences available with known dissociation constants, and out of the 136 possible single nucleotide variants, only four were not represented in these 46 sequences (Table 2). Results (Table 2 and Figure 5) are similar to those from Leu3, with the lowest energy variant usually matching the consensus, with some variants only slightly different from the lowest energy variant (e.g., C versus G at position 6), and with some variants being extremely deleterious (e.g., C at position 1).

**Table 2:** Posterior distribution of position-specific energy terms for ArcA, 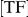 = 100 nM, average counts = 20).

	A		C		G		T	
Position	mean	std	mean	std	mean	std	mean	std
1	1.42	0.31	2.32	0.19	**0.00**	**0.00**	1.05	0.28
2	1.49	0.32	2.11	0.46	-	-	**0.00**	**0.00**
3	0.52	0.31	2.18	0.51	0.46	0.29	**0.00**	**0.02**
4	**0.00**	**0.01**	2.27	0.43	0.74	0.30	1.74	0.39
5	0.25	0.20	**0.01**	**0.05**	0.77	0.36	0.40	0.35
6	0.35	0.14	**0.06**	**0.13**	0.13	0.18	0.32	0.28
7	**0.09**	**0.15**	0.22	0.24	0.12	0.18	0.51	0.09
8	0.24	0.19	0.17	0.23	0.51	0.33	**0.03**	**0.08**
9	0.07	0.10	0.19	0.22	0.21	0.22	**0.05**	**0.10**
10	**0.02**	**0.06**	0.64	0.32	0.53	0.32	0.27	0.14
11	1.02	0.27	0.66	0.33	1.02	0.30	**0.01**	**0.05**
12	-	-	-	-	**0.00**	**0.00**	-	-
13	2.24	0.51	1.72	0.37	2.08	0.41	**0.00**	**0.00**
14	0.10	0.15	1.83	0.39	0.16	0.20	**0.03**	**0.06**
15	**0.00**	**0.01**	1.70	0.40	0.75	0.31	1.13	0.32

Notation is the same as in Table 1.

### Simulating a more complete SULDEX experiment

A more complete SULDEX experiment would include most or all of the ds-oligos that are likely to be specifically bound by a transcription factor, unlike for the Leu3 and ArcA data sets, which each have dissociation constants for fewer than 50 binding sites. Furthermore, for the Leu3 and ArcA simulations we assumed equal pre-bound frequencies, even though it is more realistic to expect variation in the relative frequencies of the different ds-oligos in the pre-bound fraction. We therefore tested our method on a more complete SULDEX experiment simulation that incorporated these factors. The best available dataset for this purpose, that we are aware of, is the Zif268 data from the Bind-n-Seq experiment [12]. The Bind-n-Seq dataset, however, presents a major practical difficulty in that the Zif268 binding sites (length 10) are located within random 21 mers, meaning that the local context for any putative binding site varies in every instance. The efficiency of ds-oligo synthesis itself can vary with local sequence context, sometimes resulting in dramatically unequal frequencies (as discussed above). Additionally, the probability that a target site is bound may vary with sequence context, and the transcription factor may even bind to different locations on a long ds-oligo. Thus, the probability of binding a sequence must be summed over all binding locations, and 10 mer frequency distributions may not directly reflect binding probabilities due to interference caused by binding at other locations. Because of these problems, one cannot expect that the true Zif268 dissociation constants values can necessarily be accurately determined from this dataset. Nevertheless, SULDEX analysis of these data can provide a large set of reasonable parameter values for simulating a dataset for the purpose of testing the method. We therefore performed such an analysis, and then simulated binding using the “known” dissociation constant values.

The first step of the simulation is the synthesis of pre-bound 10 mers at different background frequencies. Some of the frequencies in the Zif268 dataset could be used directly, but many pre-bound counts in the Zif268 dataset were low or zero. We therefore generated pre-bound sequences using a generative model (GSM; see Methods), which predicts frequencies for all ds-oligos. The next step is to simulate binding of the 10 mers according to their “known” binding affinities based on energy terms derived from the preliminary SULDEX analysis of the Zif268 data. For results below, we synthesized and analyzed 10 mers differing from the consensus by two nucleotides or fewer. Simulations were run at four transcription factor concentrations (0.1, 1.0, 10.0, and 100.0 nM) with an average of 1000, 200, 100, or 20 sequence reads per ds-oligo in the pre-bound and/or bound solutions. Bound frequencies were obtained by multiplying the pre-bound frequency by the probability of binding calculated as in Equation 2, using the GEM to obtain the binding energies and thus the 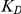
s. Simulations were replicated three times for each condition to obtain means and variance of all estimators, and were evaluated using the basic method alone (BBM), and using the basic plus GEM method, both including three known reference 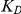
s.

**Figure 6:**
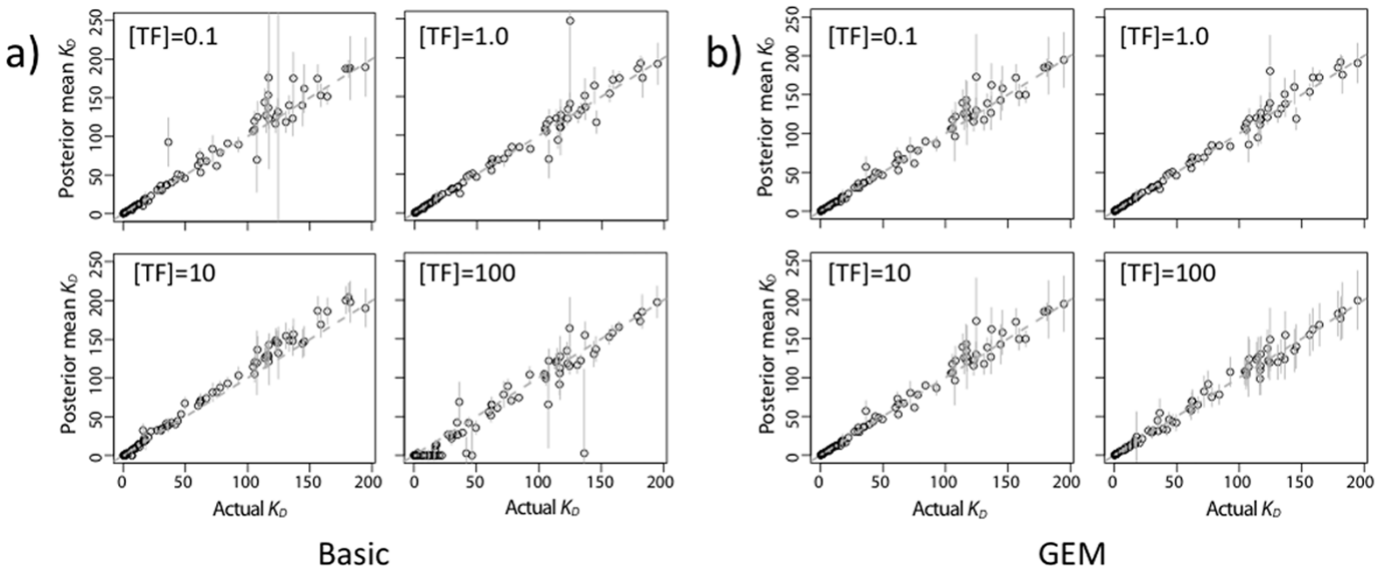
Accuracy of 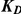 estimates for Zif268 simulation. Means and standard deviation estimates from the posterior distribution are shown for a single representative simulation replicate for each condition. In these simulations, there were 1000 counts per ds-oligo on average in both the pre-bound and bound solutions, and 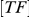 was 0.1, 1.0, 10, or 100 nM. For the Basic (BBM) model (a), but not the GEM (b) model, a reference ds-oligo with a larger 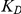 was required for 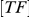 = 100 nm (c.f., Figure S1). Only the 89 high affinity ds-oligos with 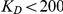 nM are shown (out of 436 total ds-oligos).

In the MCMC analysis, pre-bound frequencies and 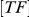
were treated as free parameters (subject to the constraints of the model) and posterior estimates of all parameters were compared to their true (simulated) values. When an average of 1000 sequence reads (counts) per ds-oligo were used, the mean 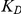 estimators for both models were generally accurate for a variety of transcription factor concentrations (Figure 6). One caveat is that the basic binding model required inclusion of a reference ds-oligo with high 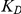 (100 nM) to obtain remotely accurate 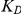 estimates when the transcription factor concentration was high (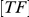 = 100 nM). In contrast, the energy model does not, and even the very lowest 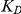
s are estimated poorly (Figure 6a, 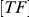 = 100 nM). Furthermore, the 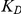 estimates were slightly worse for the basic binding method at the lowest and highest transcription factor concentrations. The transcription factor concentration estimates were accurate as well (data not shown). In general, the individual 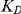 estimates were worst for ds-oligos that have very low counts in the pre-bound and/or bound fractions.

**Figure 7:**
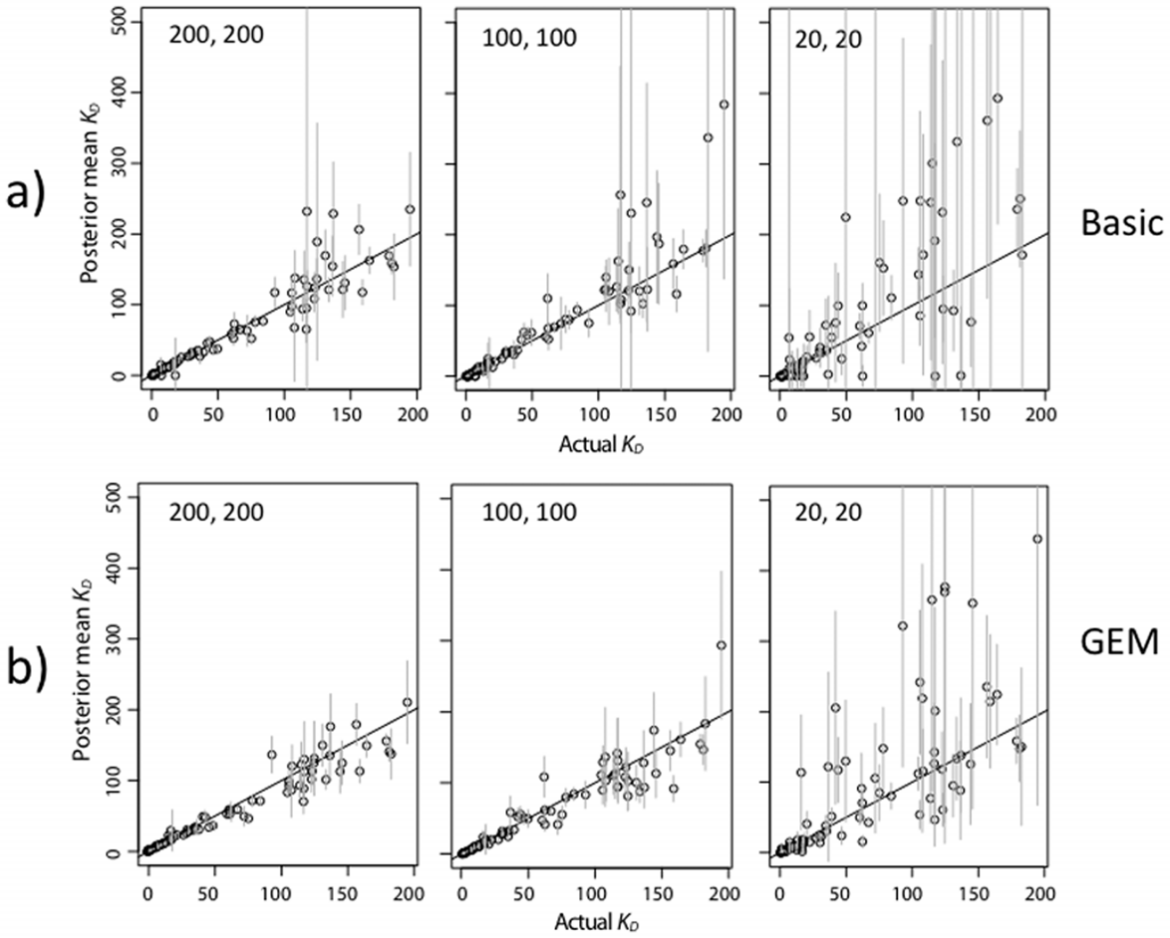
Accuracy of Zif268 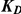 estimates with fewer counts in both solutions. Results are displayed as in Figure 6. The 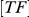 was 1.0 nM and there were 200, 100, or 20 sequence counts per ds-oligo in both the both the pre-bound and bound solutions (labeled as “pre-bound, bound” in the different sub-figures).

**Figure 8:**
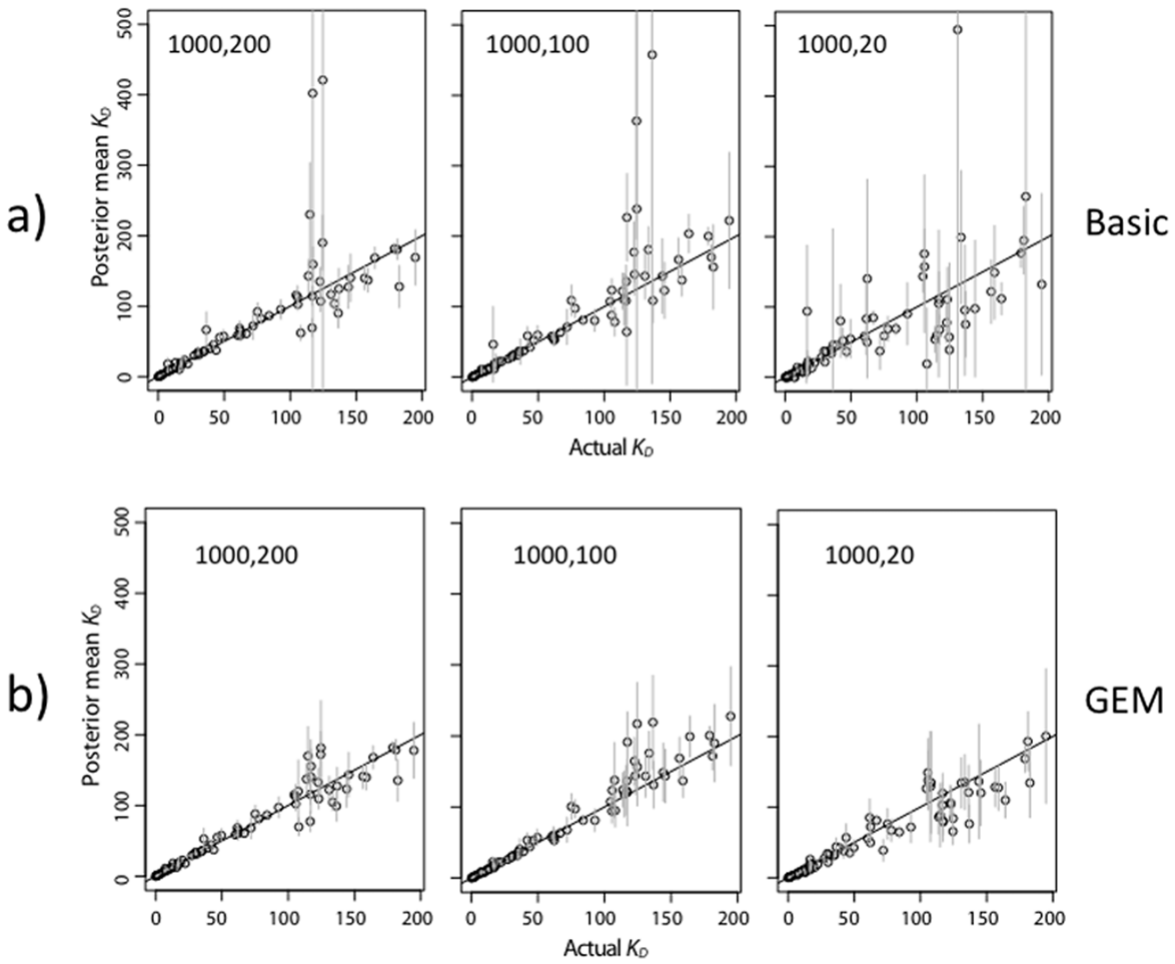
Accuracy of Zif268 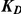 estimates with fewer counts in bound solution only. Results are displayed as in Figure 6. The 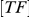 was 1.0 nM, and there were 1000 counts per ds-oligo in the pre-bound solutions. There were 200, 100, or 20 sequence counts per ds-oligo in the bound solutions. Pre-bound and bound solution concentration are labeled in the different sub-figures as in Figure 7.

**Figure 9:**
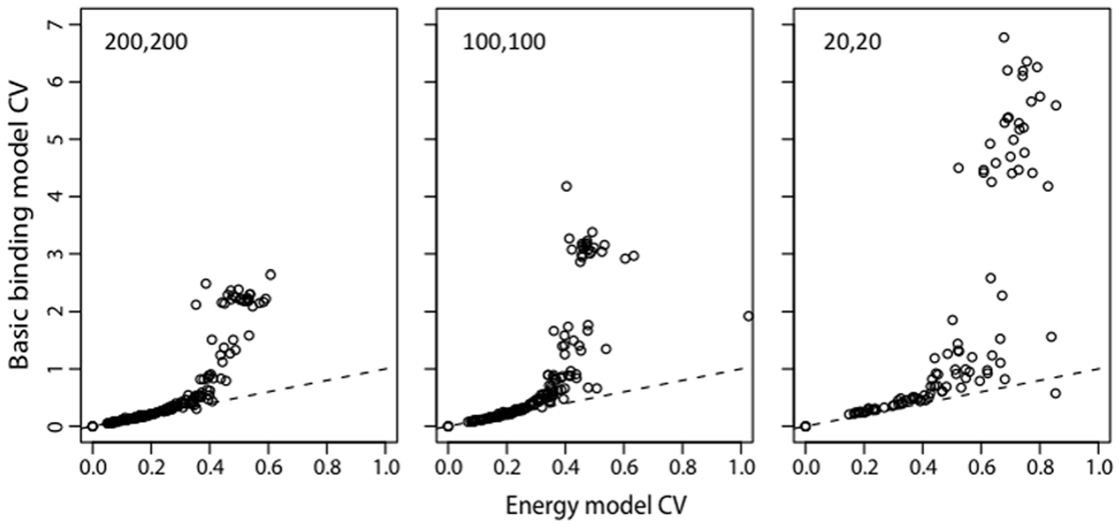
Relationship of BBM and GEM coefficients of variation for Zif268 simulations. Coefficients of variation (CVs, standard deviations over the means) were calculated from the posteriors using the same data as shown in Figure 7. The dashed line indicates equal CVs for the BBM and GEM estimators.

**Figure 10:**
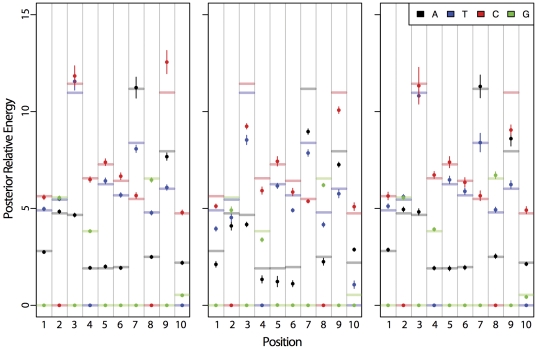
Posterior estimates of nucleotide- and position-specific energy terms. The 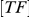 was 1.0 nM, and counts per ds-oligo in the pre-bound and bound solutions were 1000 and 1000, 100 and 100, or 1000 and 100, as labeled on the sub-figures. Results for nucleotide A, T, C, and G energy terms at each position are color coded black, blue, red, and green, respectively. The mean posterior estimate is shown with a dot, the 95% credible regions of the estimates are shown with vertical bars, and the true values are shown with horizontal bars.

As described in the previous section, the energy model is expected to provide a greater benefit when more counts in the pre-bound or bound fraction are low. This prediction is borne out by the simulations with reduced sequencing per ds-oligo (Figure 7 and 8). The GEM has better accuracy, particularly for ds-oligos with high 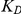 values (Figure 9), indicating that it provides a better estimator by sharing information across sequences according to the GEM. The effect is even more pronounced when the pre-bound ds-oligos are sequenced in higher numbers, but the bound ds-oligos are not. The GEM energy terms themselves are also estimated accurately as long as the pre-bound sequencing is high (Figure 10), although some of the largest energy terms are estimated less accurately than the others. This is to be expected, since the high-energy terms lead to ds-oligos with weak binding properties for which the strength of binding can only be approximately estimated.

## Discussion

The core principle applied in this study is that in solution and at equilibrium, the proportion of a particular TFBS sequence that is bound to a transcription factor (the probability of binding) is determined by the dissociation constant between the sequence and transcription factor, and the concentration of free transcription factor. Therefore, if one knows the relative frequencies of different sequences in a mixture prior to binding, one can predict the relative frequencies of those sequences that are bound to the transcription factor. A difficulty arises, in that the free concentration of transcription factor is generally unknown, and if the equilibrium constants are also unknown then there are more unknowns than data points (i.e., ds-oligo sequence counts in the bound solution). This problem can be easily solved by including at least two reference sequences with known dissociation constants in the basic (BBM) system, and if an energy model (GEM) is used, only one reference sequence is required. To ensure accuracy, it is also best if the counts for the reference sequences are relatively high. Although one could first solve for the unknowns using the reference sequence data, we have chosen to use a flexible Bayesian approach that can account for errors and uncertainty in all data points and allows inclusion of as many reference sequences as are available.

One of the main benefits of the Bayesian approach is that it can incorporate information from multiple sources, all with varying degrees of uncertainty. In the simple graphical system that does not model the binding energy (Figure 1), the sources of information are the counts of sequences before the transcription factor is bound (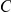) and the counts in the fraction bound to the transcription factor (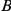). It is assumed that these counts are random draws from a multinomial based on the true underlying proportions of the sequences in the two solutions, respectively 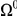 and 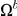. These counts thus constrain the reasonable range of values for the underlying proportions, with higher counts constraining the proportions more than lower counts. The ds-oligo proportions in the bound solution containing transcription factor are modified from the pre-bound proportions based on the sequence-specific probability of binding the transcription factor. The reference sequences, with known dissociation constants, allow the information about the proportions of sequences in the two solutions to be translated back into information about the dissociation constants, and thus the binding energy of each sequence 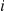 through the relationship 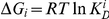 (see Methods for details). Inclusion of at least two reference sequences ensures the identifiability of all unknown parameters.

An interesting illustration of the flexibility of our Bayesian approach is our use of “generative energy models” (GEMs). With these models, information is communicated across ds-oligo sequences to predict the binding energy. In GEMs, the binding energy is considered to be composed of independent position-specific energy components, as well as interaction energy components [13]. The energy model may not be perfectly accurate, however, and the error term for the energy model allows for automated weighting of the accuracy of the energy-based predictions and the relative frequency-based predictions in coming to a joint posterior prediction of each sequence-specific dissociation constant. This weighting allows an easy switch from a basic method that relies on reference ds-oligos with known 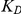
s and the counts alone, if they are high enough, to the GEM portion of the model, if the error in the GEM portion is low enough.

There are similarities between the GEM and a position weight matrix (PWM) model using binding energies since both are (typically) estimated using independent energy contributions for each position [40]. The main difference is that in PWM estimation, the free 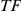 concentration is assumed to be low. If this assumption is inaccurate the PWM will return an incorrect energy model. Therefore, an advantage of approaches such as ours that allow 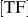 to vary, and to be estimated in the model-fitting process (see also [13]), is that a more accurate energy model can be determined without restrictive assumptions. In addition, our method also contrasts with PWM fitting in the use of reversible jumps between models of different dimensions to allow simultaneous estimates of the parameters of interest (the binding energies) while only including those energy components that are statistically well-justified.

We have also proposed a separate “generative sequence model”, or GSM, which allows us to use the frequencies of shorter sub-sequences (polynucleotides) to model the frequency of sequences in solution prior to binding. This is useful in cases (such as the Zif268 Bind-n-Seq data) where there are so many pre-bound sequences that most of them individually have extremely low frequencies and are therefore difficult to count. For example, as we showed for the Bind-n-Seq data [12], the relative frequencies of their “randomly”-generated ds-oligos of even 8 bp in length can differ by many orders of magnitude. The range of frequencies for longer ds-oligos is even greater, and thus if accuracy is a concern it may be impossible to obtain sufficient coverage of all ds-oligos in a highly complex mixture. The sequencing requirements for accurate frequency estimates can easily be far beyond the capacity of even the best modern high-throughput sequencers. The GSM ameliorates this problem to some extent by providing improved frequency estimators for ds-oligos with low sequence counts, and may be further improved by discovering and incorporating other general rules to predict frequency variation. In the meantime, careful experimental design to reduce the number of sequences in the mixture and focus on particular experimental questions is also important if accuracy is a concern.

Two recently proposed approaches similar to the SULDEX experimental system, Bind-n-Seq [12] and HT-SELEX [13], also utilize ds-oligos sequenced before and after binding to a transcription factor. The Bind-n-Seq analysis was designed to qualitatively identify binding site preferences, and assumes a simple PWM model with no attempt to model the biophysical energy terms or the relationship between the pre-bound and bound solution counts. As we have noted here, the experimental results are suboptimal for the purpose of measuring binding energies primarily because binding sites are created randomly in the varying context of a much longer random ds-oligonucleotide. This variable context can affect the probability of ds-oligo synthesis and the probability of binding, adding noise and bias to the results. The HT-SELEX approach, in contrast, has a similar aim to the SULDEX approach, and uses the BEEML program to estimate transcription factor concentrations and energy terms using a maximum likelihood approach and a nearly identical biophysical model. Key advantageous features of our approach include: 1) SULDEX uses a Bayesian approach and MCMC rather than a maximum likelihood approach, resulting in posterior distributions and an automatic estimate of uncertainty rather than only a point estimate. Bayesian and MCMC approaches based on biophysical models of transcription factor binding have previously been used to analyze ChIP-chip or PBM data [48], [61], and are particularly useful for parameter-rich models such as these; 2) Unlike HT-SELEX, SULDEX allows estimation of binding energies directly from relative frequencies or in combination with an energy model, and allows incorporation of reference sequences with known binding energies. Thus, while both approaches include an energy model error term, in SULDEX this term allows reduced dependence on the energy model assumptions when binding energies are inferred. Binding energy inference will flexibly depend more on the energy model for low frequency ds-oligos with insufficient sequencing to be estimated by the BBM approach alone; 3) SULDEX uses the multinomial distribution to calculate the probabilities of observed counts given underlying ds-oligo frequencies in solution, while HT-SELEX's BEEML program uses a Gaussian approximation. This is most likely to matter when some counts are small, which will often occur due to the high variance of starting ds-oligo frequencies. BEEML also uses energy level discretization to approximate the partition function; 4) We have presented here a model (GSM) to predict relative ds-oligo frequencies in the pre-bound fraction, and shown that the simple independent nucleotide frequency model used in HT-SELEX/BEEML can be highly inaccurate. The GSM should be further developed to increase accuracy when data from a larger number of pre-bound ds-oligo synthesis experiments become available; 5) the SULDEX method allows simultaneous inference of model complexity and parameter values by incorporating a reversible jump MCMC approach, thus avoiding potential problems of model over-specification that can lead maximum likelihood estimators (such as those in BEEML) to focus on noise and thus reduce prediction accuracy.

We expect that a future application of this and related approaches will be to allow detailed study of how binding energies are influenced by nucleotide variation in the binding site. In particular, the simple additive energy model is insufficient to accurately predict dissociation constants for many proteins, and interactive energy terms will therefore be needed. The number of possible interactive energy terms can be quite large, and their statistical justification and usefulness should be carefully considered. The reversible jump MCMC approach applied here can be used for this purpose and allows detailed biophysical models to be developed, evaluated, and compared without making a large number of assumptions. However, to explore interaction terms, it will be important to implement careful experimental design for generating data. For the datasets currently available (Bind-n-Seq and HT-SELEX), we do not believe that the pre-bound frequencies can be reliably determined, and for the Bind-n-Seq data the binding probabilities are confounded by variable context and possible multiple binding opportunities per ds-oligo. Thus, it seems quite possible that estimation of higher-order interactions in these datasets could be thoroughly confounded by these other effects. For example, in our own focused experiments using mitochondrial transcription factor A protein (mtTFA; unpublished data), which has moderate sequence specificity [62], we found that its multiple binding modes and multimers that form on the ds-oligos, as well as the context of varying sequence well outside the binding site, can deeply confound interpretation. Experiments that therefore focus on a sub-sample of possible ds-oligos close to the consensus or optimal ds-oligo can produce precise estimates of binding energies without relying on a model, and these may then be used to better elucidate reasonable model structures.

Characterizing the binding potential of target binding sites for a transcription factor within a species has immediate benefit for understanding transcriptional regulation for a particular system, but applying this strategy across multiple species may have more widespread impact. In particular, to understand morphological evolution, it is necessary to have a clear idea of the relationship between transcription factors, their strength of binding to a wide range of targets, and how the binding energy relationships change as transcription factors evolve. Future implementations can also include important factors such as cooperative binding and interaction with repressors. We therefore expect that exploiting methods for determining binding energies across species will lead to substantial impact in other fields and in understanding to approach questions relating to disease pathology, evolutionary adaptation, and speciation.

## Supporting Information

Figure S1
**True versus predicted binding with and without inclusion of an energy model for Leu3.** The relationship between the true and predicted log 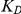
s are shown in (a), with results that included the GEM model and an error term shown with red circles, and results without an energy term (the BBM model) with blue circles. The posterior distribution of the respective error terms for the GEM model results are shown in (b). Results are shown for free 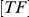 of 10 nM (labeled a1 and a3, and b1 and b3) or 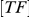 = 50 nM (labeled a2 and a4, and b2 and b4). Average counts per ds-oligo were also varied, with counts of 10 in a1 and a3 (and b1 and b3), and counts of 100 in a2 and a4 (and b2 and b4). Dotted lines represent perfectly accurate 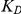 predictions.(PDF)

Figure S2
**True versus predicted binding with and without inclusion of an energy model for ArcA.** Results shown for ArcA are the same as for Leu3 in Figure S2, except that free 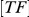 was 100 nM or 1000 nM (1 µM).(PDF)
