# Supplementary figures and images for: Bayesian Analysis of High-Throughput Quantitative Measurement of Protein-DNA Interactions
Source: PLoS One. 2011 Nov 1;6(11):e26105. doi: 10.1371/journal.pone.0026105 (PMC3206046; doi:10.1371/journal.pone.0026105)

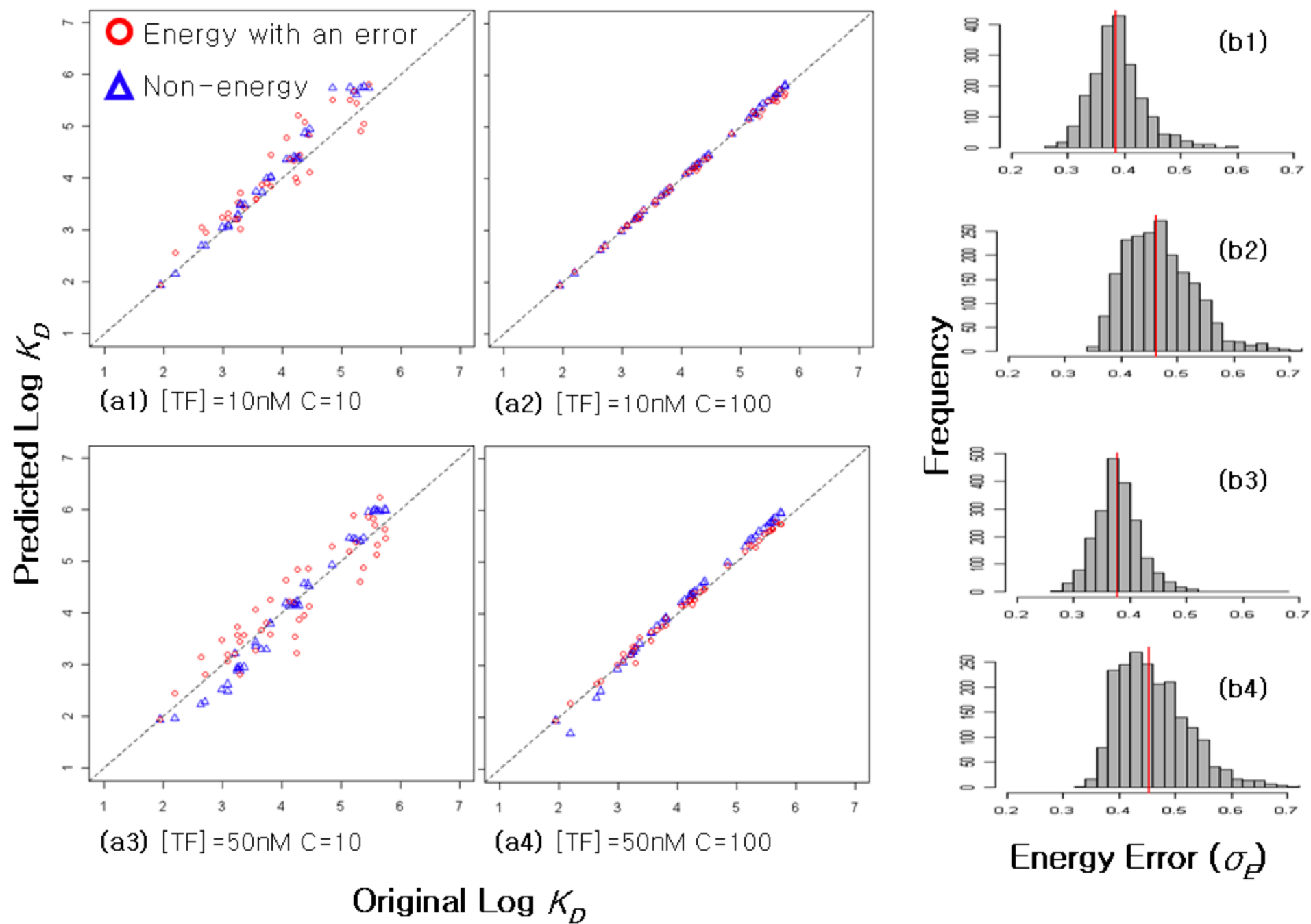

**Figure S1**

Supplement: Figure S1 — True versus predicted binding with and without inclusion of an energy model for Leu3. The relationship between the true and predicted log s are shown in (a), with results that included the GEM model and an error term shown with red circles, and results without an energy term (the BBM model) with blue circles. The posterior distribution of the respective error terms for the GEM model results are shown in (b). Results are shown for free of 10 nM (labeled a1 and a3, and b1 and b3) or = 50 nM (labeled a2 and a4, and b2 and b4). Average counts per ds-oligo were also varied, with counts of 10 in a1 and a3 (and b1 and b3), and counts of 100 in a2 and a4 (and b2 and b4). Dotted lines represent perfectly accurate predictions. (PDF) [file pone.0026105.s001.pdf]

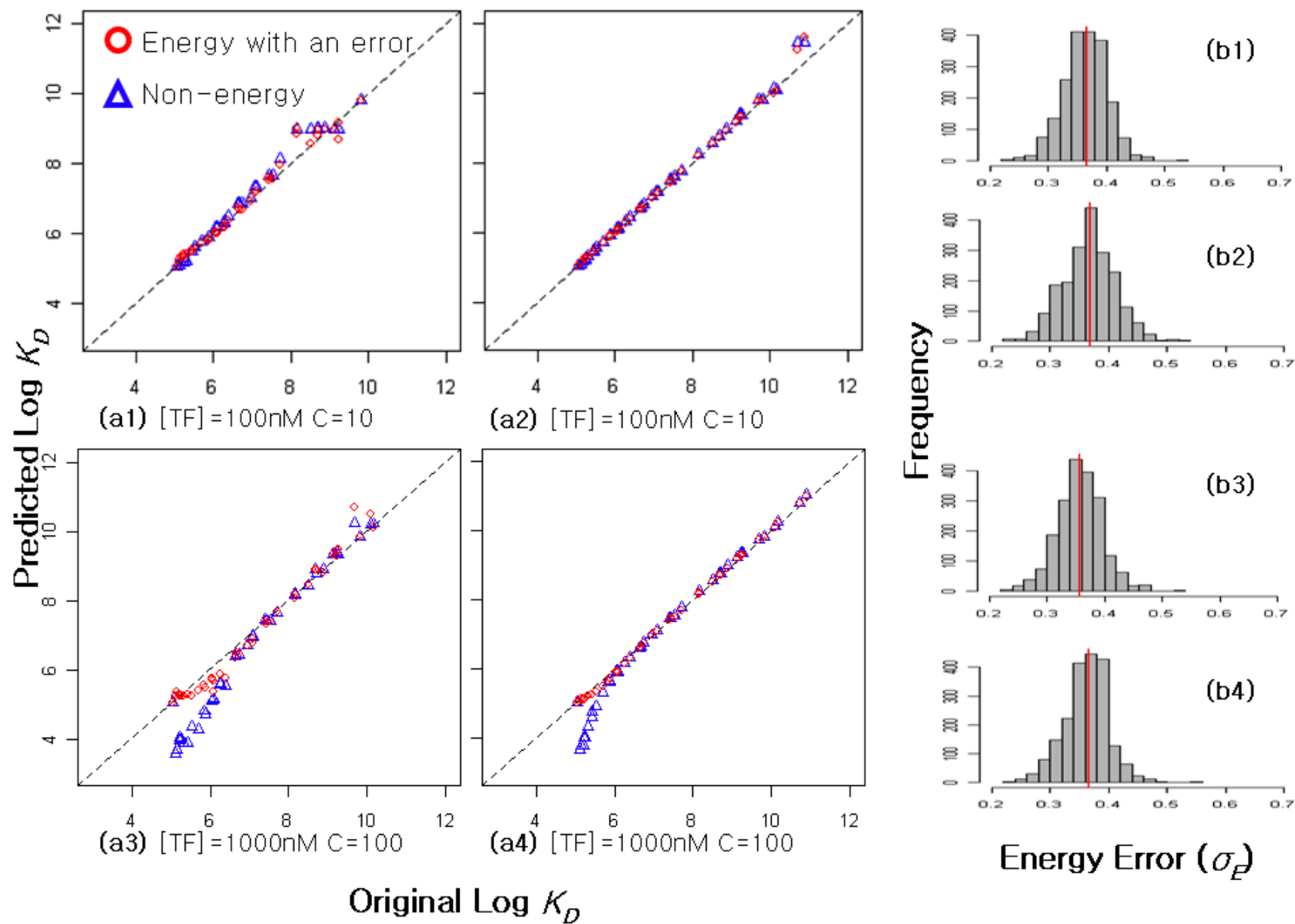

**Figure S2**

Supplement: Figure S2 — True versus predicted binding with and without inclusion of an energy model for ArcA. Results shown for ArcA are the same as for Leu3 in Figure S2, except that free was 100 nM or 1000 nM (1 µM). (PDF) [file pone.0026105.s002.pdf]
